# Herpes zoster is associated with an increased risk of subsequent lymphoid malignancies - A nationwide population-based matched-control study in Taiwan

**DOI:** 10.1186/1471-2407-12-503

**Published:** 2012-10-31

**Authors:** Yi-Chang Liu, Yi-Hsin Yang, Hui-Hua Hsiao, Wen-Chi Yang, Ta-Chih Liu, Chao-Sung Chang, Ming-Yu Yang, Pai-Mei Lin, Jui-Feng Hsu, Pi-Yu Chang, Sheng-Fung Lin

**Affiliations:** 1 Division of Hematology-Oncology, Department of Internal Medicine, Kaohsiung Medical University Hospital, 100, Tzyou 1st Road, Kaohsiung 807, Taiwan; 2 Department of Internal Medicine, Faculty of Medicine, College of Medicine, Kaohsiung Medical University, 100, Tzyou 1st Road, Kaohsiung 807, Taiwan; 3 Graduate Institute of Medicine, College of Medicine, Kaohsiung Medical University, 100, Tzyou 1st Road, Kaohsiung, 807, Taiwan; 4 Statistical Analysis Laboratory, Department of Medical Research, Kaohsiung Medical University Hospital, 100, Tzyou 1st Road, Kaohsiung, 807, Taiwan; 5 Graduate Institute of Clinical Medical Sciences, College of Medicine, Chang Gung University, Wen-Hwa 1st Road, Kwei-Shan, Tao-Yuan, 333, Taiwan; 6 Department of Nursing, I-Shou University, 1 ,Sec. 1, Syuecheng Road., Dashu District, Kaohsiung, 840, Taiwan

**Keywords:** Herpes zoster, Lymphoma, Leukemia, Epidemiology, Taiwan

## Abstract

**Background:**

Infectious agents have been shown to contribute to the development of lymphoid malignancies. The different distribution of lymphoid malignancies in Asian and Western populations suggests possibly different etiologies in Asian populations. Herpes zoster infection, commonly seen in immunocompromised persons, has been reported to be associated with lymphoid malignancies in retrospective case–control studies from Western populations, but the results are controversial and large-scale prospective studies from Asian populations are lacking.

**Methods:**

A nationwide population-based matched-controlled prospective study on Taiwanese patients was performed using the National Health Insurance Research Database from 1996 to 2007. Herpes zoster and malignancies were defined by compatible ICD-9-CM (International Classification of Disease, 9^th^ Revision, Clinical Modification) codes. Patients who had been diagnosed with any malignancies before herpes zoster, with known viral infections including human immunodeficiency virus, and duration from herpes zoster to diagnosis of malignancies less than 6 months were excluded.

**Results:**

Of 42,498 patients with herpes zoster prior to the diagnosis of any malignancies, the cumulative incidence for lymphoid malignancies was 0.11% (n = 48), compared with 0.06% (n = 106) in 169,983 age- and gender-matched controls (univariate hazard ratio (HR): 1.82, 95%CI: 1.29-2.55). The most common lymphoid malignancy was non-Hodgkin’s lymphoma (60.4%, n = 29), followed by multiple myeloma (27.1%, n = 13). Risk for developing lymphoid malignancies is significantly higher in herpes zoster patients (log rank *P* = 0.005). After adjusting for presence of any comorbidities in Charlson comorbidity index, time-dependent covariate for herpes group, and income category using Cox proportional hazard regressions, herpes zoster patients had an increased risk of developing lymphoid malignancies (adjusted HR: 1.68, 95%CI: 1.35-2.42, *P* = 0.0026), but did not have an increased risk of developing non-lymphoid malignancies (adjusted HR: 1.00, 95%CI: 0.91-1.05, *P* = 0.872).

**Conclusion:**

Preceding herpes zoster infection is an independent risk marker for subsequent lymphoid malignancies in Taiwanese subjects. Further studies are warranted for pathogenesis exploration and preventive strategies in Asian populations.

## Background

Lymphoid malignancies are characterized by the malignant transformation of lymphoid cells and comprise a group of heterogeneous diseases with distinctive clinical, immunophenotypic and genetic features. The etiologies remain poorly understood, but diseases that impair cellular immunity such as human immunodeficiency virus (HIV) infection and other immunodeficient diseases significantly predispose to lymphoid malignancies [1-4]. Some host factors, including inherited genetic factors, infections, autoimmune diseases, and environmental or medication exposure have been found to be associated with the development of lymphoid malignancies [4]. Recognition of the role of host infections in lymphomagenesis is important for prevention and management strategies. Some viral infections have been found to be associated with the development of certain lymphoid malignancies, such as Epstein-Barr virus (EBV) and Burkitt’s lymphoma, extranodal NK/T cell lymphoma, human T-cell leukemia virus type 1 (HTLV-1) and adult T cell leukemia/lymphoma, human herpes virus-8 (HHV-8) and primary effusion lymphoma, and hepatitis C virus (HCV) and some B cell lymphomas [4-7]. However, the association between herpes zoster and subsequent lymphoid malignancies is still unclear.

Herpes zoster is typically characterized by unilateral crops of painful and pruritic vesicles in a dermatomal distribution, and is caused by reactivation of latent varicella-zoster virus (VZV). The VZV establishes latency in the dorsal root ganglia and its reactivation is associated with a decline in cell-mediated immunity [8,9]. The decline of VZV-specific cell-mediated immunity may be either a natural consequence of aging or as a result of immunosuppression [10]. Since impairment of immunity is often linked with carcinogenesis, there have been studies exploring the preceding herpes zoster infection as an indicator of subsequent cancer. Although most of these studies indicated no obvious increase risk of subsequent cancer [11,12], an increased risk of developing lymphoid malignancies was found from several population-based case–control studies in patients with history of herpes zoster infection, including chronic lymphocytic leukemia (CLL) [13,14], multiple myeloma or monoclonal gammopathy of undetermined significance [15,16], and lymphoplasmacytic lymphoma or Waldenstrom macroglobulinemia [17].

The distribution of lymphoid malignancies in Asia is different from that in Western countries [18-22], indicating racial and geographic differences in the etiologies of lymphoid malignancies. Recognition of preceding factors prior to lymphoid malignancies, especially infectious agents, is important to understand the pathogenesis and the distribution differences. However, little is known about the association between herpes zoster and subsequent risk of lymphoid malignancies in Asian populations, as most case–control studies are retrospective design from Western populations and large-scale prospective studies from Asian populations are lacking. Therefore, we performed a matched-control prospective follow-up study from a nationwide population-based dataset in Taiwan and tried to explore the association between herpes zoster exposure and the subsequent risk of lymphoid malignancies.

## Methods

### Data source

This study used the National Health Insurance Research Database (NHIRD) from 1996 to 2007, which is derived from the Taiwan National Health Insurance (NHI) program and provides a sample of 1,000,000 random subjects to scientists in Taiwan for research purposes. The NHI program has been implemented in Taiwan since 1995, offering a comprehensive, unified, and universal health insurance program to all citizens who have established a registered domicile for at least four months in the Taiwan area. The coverage rate was 96% of the whole population in 2000 rising to 98.4% (22.6 million of the country’s 22.96 million people) at the end of 2007. The coverage provides outpatient services, inpatient care, Chinese medicine, dental care, childbirth, physical therapy, preventive health care, home care, and rehabilitation for chronic mental illnesses. The NHI medical claims database includes ambulatory care, hospital inpatient care, dental services, and prescription drugs. Therefore, the NHIRD is the largest and most complete nationwide population-based dataset in Taiwan, and there are no statistically significant differences in age, sex, and average insured payroll-related amount between the sample group and all enrollees. These features make the dataset a valuable resource for examining the risk of developing lymphoid malignancies among patients with herpes zoster. Because the NHIRD database provided by the official NHI program consists of totally de-identified, encrypted, secondary data released to the public for research purposes without personal or institutional identification or contact with the study patients, the study was exempt from full review by the institutional review board (IRB) of Kaohsiung Medical University. The study also conformed to the criteria of exemption of IRB review and exemption of obtaining informed consents which were announced by the Department of Health.

### Study sample

We selected all patients who had visited ambulatory care centers for the treatment of herpes zoster between 1996 and 2007. Only patients who had the first herpes zoster exposure were selected. We defined herpes zoster by compatible ICD-9-CM (International Classification of Disease, 9^th^ Revision, Clinical Modification) codes of herpes zoster (053.0-053.9) on at least one service claim for inpatient or outpatient care. The definition of lymphoid malignancies was derived from the 2008 WHO classification of tumours of hematopoietic and lymphoid tissues [1], and cases were identified by compatible ICD-9-CM codes including Hodgkin’s disease (code 201.0-201.9), non-Hodgkin’s lymphoma (code 200.0-200.8, 202.0-202.9), multiple myeloma (code 203.0-203.1), and lymphoid leukemia (code 204.0-204.9). We excluded patients who had been diagnosed with any lymphoid malignancies or any cancers (code 140.0-199.1) before herpes zoster. Furthermore, we excluded patients who had been diagnosed with other viral infections (code 045.0-052.9, 054.0-066.9, 070–079.9) and HIV infection (code 042) before the diagnosis of lymphoid malignancies. Patients with their duration from herpes zoster to diagnosis of any malignancies less than 6 months were also excluded. In total, 42,498 patients with herpes zoster were included in the study group. The first ambulatory care visit for the treatment of herpes zoster was assigned as the index visit.

The matched-control group was likewise extracted from the Registry of Beneficiaries of the NHIRD. We randomly selected 169,983 control subjects (4 for every herpes zoster patient), matched with the study group in terms of age, sex, and the year and month of index visit. After matching with age and sex, the month of index visit of the matched cases was assigned the same as the index visit of study group, and follow-up started 6 months after the index visit. Similar to the study group, patients who had lymphoid malignancies or any cancers before their index ambulatory care visit, who had other viral infections (code 045.0-052.9, 054.0-066.9, 070–079.9) and HIV infection (code 042) before the diagnosis of lymphoid malignancies, and patients with an interval from index visit to the diagnosis of any malignancies of less than 6 months were excluded in the control group.

### Statistical analysis

The chi-square test was used to compare the distribution of demographic characteristics between patients with and without herpes zoster. Time-to-event analysis involved estimating the probability that lymphoid malignancies would occur at different points in time. The end-point of follow-up in those who developed lymphoid malignancies was the date of diagnosis, and in those who did not develop lymphoid malignancies was the end of observation (December 31, 2007), to arrive at “censored” data. Kaplan-Meier estimates were computed to compare the difference in developing lymphoid malignancies between patients with and without herpes zoster.

The univariate and multivariate proportional hazards model was applied to estimate the risk effect on developing lymphoid malignancies and all malignancies (excluded lymphoid malignancies). The assessment of comorbidities was performed in the year before the index visit and was integrated into multivariate analysis model. One of the most commonly used comorbidities index to rate the impact on index disease in various medical researches is Charlson comorbidity index (CCI) [23-25]. The CCI was developed by assigning weights for 19 chronic conditions, taking into account of the number and seriousness of comorbid diseases. The covariates in the analysis model included age, sex, income category (monthly income ≦USD 800; monthly income >USD 800), time-dependent covariate for herpes zoster group, and existence of any chronic conditions listed in the CCI. To exclude the possibility that the adjusted hazard ratio (HR) of herpes zoster is influenced by any comorbidities, each adjusted HR for herpes zoster was calculated from different models by adjusting all comorbidities, and by adjusting comorbidities with step-wise removal of one comorbidity at one time. All statistics were calculated using SAS 9.2 software. All P values are two-sided.

## Results

The demographic characteristics and comorbid medical disorders of the patients with herpes zoster and the control group matched in terms of age, sex and the year and month of index visit are shown in Table 1. The comorbidities were derived from the items listed in the CCI. A total of 42,498 patients were found to have herpes zoster prior to the diagnosis of any malignancies. The mean age was 48.92 years (± 20.67 years), with a mild female predominance (52.4%). The demographic characteristics between the patients and controls were largely different in distribution. In general, herpes zoster patients had more comorbidities than controls, except for myocardial infarction, dementia, hemiplegia or paraplegia, and moderate or severe liver disease (Table 1).

**Table 1:** Demographic characteristics and comorbid medical disorders in patients with herpes zoster and the matched-control cohort

**Variable**	**Patients with herpes zoster**	**Matched**- **control subjects**	**Odds ratio**	**95**% **CI**	***P*value**
Total	42,498	169,983			
Follow-up			-	-	-
Total person-years	168,992	684,001			
Average person-years	3.976	4.024			
Sex			-	-	0.9874
Female	52.4	52.4			
Male	47.6	47.6			
Age, years-old			-	-	1.0000
>= 60	40.0	40.0			
50-59	19.6	19.6			
40-49	12.9	12.9			
30-39	10.7	10.7			
< 30	16.6	16.6			
Comorbidities (%)					
Myocardial infarct	1.4	1.2	1.12	0.95-1.33	0.1846
Congestive heart failure	4.6	3.7	1.28	1.18-1.39	<0.0001
Peripheral vascular disease	2.0	1.6	1.32	1.15-1.51	<0.0001
Cerebrovascular disease	10.5	9.5	1.19	1.13-1.25	<0.0001
Dementia	1.3	1.4	1.12	0.99-1.27	0.0667
Chronic pulmonary disease	29.6	24.6	1.38	1.33-1.43	<0.0001
Rheumatologic disease	4.2	2.9	1.92	1.74-2.10	<0.0001
Peptic ulcer disease	25.7	21.2	1.40	1.35-1.45	<0.0001
Mild liver disease	18.0	15.0	1.33	1.27-1.39	<0.0001
Diabetes (mild to moderate)	13.3	11.1	1.33	1.28-1.39	<0.0001
Diabetes (with chronic complications)	4.0	3.2	1.36	1.26-1.47	<0.0001
Hemiplegia or paraplegia	1.0	1.1	1.13	0.94-1.36	0.1897
Renal disease	6.0	4.8	1.43	1.33-1.54	<0.0001
Moderate or severe liver disease	0.4	0.4	1.09	0.82-1.45	0.9568

**Figure 1:**
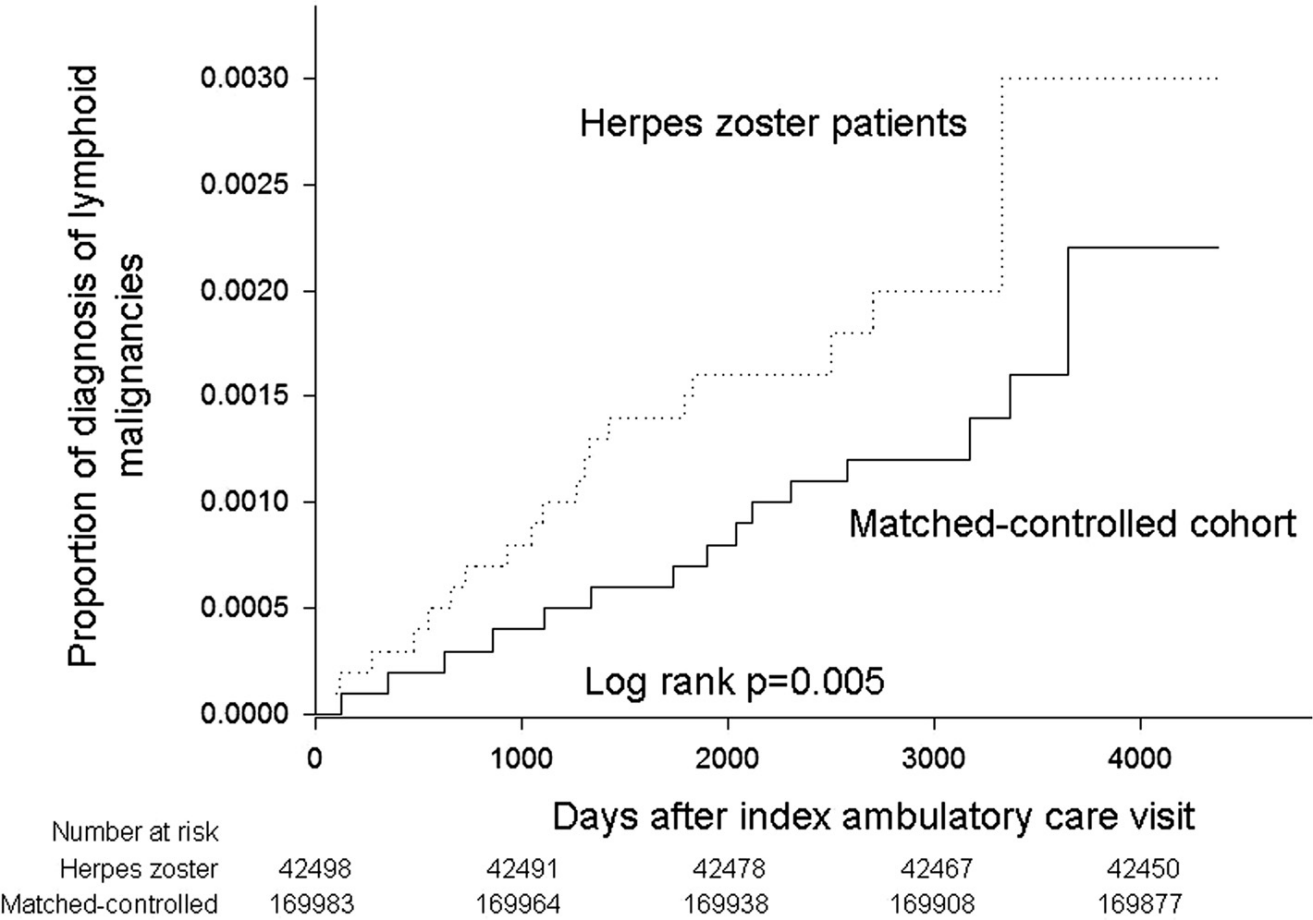
**The cumulative incidence of lymphoid malignancies among patients with herpes zoster and matched**-**controlled cohort.** The Kaplan-Meier estimates of the cumulative incidence of lymphoid malignancies showed the risk for developing lymphoid malignancies is significantly higher in herpes zoster patients than in the matched-controlled cohort (log rank p = 0.005).

The proportion of those developing lymphoid malignancies or any cancers (excluding lymphoid malignancies) of the patients and controls is shown in Table 2. Among the patients with herpes zoster, 2.42% (n = 1,027) subsequently developed cancer, and 0.11% (n = 48) lymphoid malignancy. Among the controls, 2.26% (n = 3,838) of the patients subsequently developed cancer, and 0.06% (n = 106) lymphoid malignancy. Among 48 herpes zoster infected patients who developed subsequent lymphoid malignancies, the most common lymphoid malignancy was non-Hodgkin’s lymphoma (60.4%, n = 29), followed by multiple myeloma (27.1%, n = 13). Patients with herpes zoster had a significantly increased risk of developing any cancers (excluding lymphoid malignancies, univariate HR: 1.07, 95% CI: 1.01-1.15), and lymphoid malignancies (univariate HR: 1.82, 95% CI: 1.29-2.55) compared with the control group. The Kaplan-Meier estimates of the cumulative incidence of lymphoid malignancies among the patients with herpes zoster and the controls are shown in Figure 1. Risk for developing lymphoid malignancies is significantly higher in herpes zoster patients than in the matched-controlled cohort (log rank p = 0.005).

**Table 2:** Univariate HRs for lymphoid malignancy and all malignancies (excluding lymphoid malignancy) among patients and controls

	**Total**	**Patients with herpes zoster**	**Matched**-**control subjects**
Presence of lymphoid malignancy			
Yes, no. (%) of patients	154 (0.07)	48 (0.11)	106 (0.06)
No, no. (%) of patients	212,105 (99.93)	42,348 (99.89)	169,757 (99.94)
Univariate HR^a^ (95% CI)	-	1.82 (1.29-2.55)	Reference
*P* value		0.0006	
Presence of cancer (excluded lymphoid malignancy)			
Yes, no. (%) of patients	4,865 (2.29)	1,027 (2.42)	3,838 (2.26)
No, no. (%) of patients	207,590 (97.71)	41,458 (97.58)	166,132 (97.74)
Univariate HR^a^ (95% CI)	-	1.07 (1.01-1.15)	Reference
*P* value		0.0465	

^a^HR: Hazard ratio.

To adjust for potentially confounding factors and survival bias, covariates of CCI, income category, and time-dependent covariate for herpes zoster group were added to Cox proportional hazard regressions. After adjusting for existence of any comorbidities listed in the CCI and income category, patients with herpes zoster had an increased risk of developing lymphoid malignancies (adjusted HR: 1.68, 95% CI: 1.35-2.42, *P* = 0.0026), but did not have an increased risk of developing non-lymphoid malignancies (adjusted HR: 1.00, 95% CI: 0.91-1.05, *P* = 0.872) (Table 3). To exclude the possibility that the adjusted HR is influenced by any comorbidities, each adjusted HR for herpes zoster was calculated from different models by adjusting all comorbidities and by adjusting comorbidities with step-wise removal of one comorbidity at one time. The adjusted HR for herpes zoster showed similar value from different models (1.67-1.76, Additional file 1: Table S1), indicating that the influence of any comorbidities on the adjusted HR is low, and herpes zoster is an independent risk marker for subsequent lymphoid malignancies.

**Table 3:** Adjusted hazard ratios for lymphoid malignancy and all malignancies (excluding lymphoid malignancy)

**Variable**		**Adjusted HR**^**a**^**for lymphoid malignancy** (**95%CI**)	***P*value**	**Adjusted HR**^**a**^**for all malignancies** (**95%CI**)	***P*value**
Herpes zoster					
	No	Reference		Reference	
	Yes	1.68 (1.35-2.42)	0.0026	1.00 (0.91-1.05)	0.872
Charlson comorbidity index^b^					
	No	Reference		Reference	
	Yes	2.33 (1.66-3.27)	<0.0001	2.92 (2.75-3.11)	<0.0001
Income category (monthly)					
	≤ USD 800	Reference		Reference	
	> USD 800	0.58 (0.36-0.93)	0.023	0.63 (0.58-0.68)	<0.0001

^a^HR: Hazard ratio. Adjusted by Charlson comorbidity index, time-dependent covariate for herpes zoster group, and income category.

^b^Presence of any comorbidities listed in Charlson comorbidity index.

## Discussion

This study is a large population-based matched-control prospective follow-up survey on a Taiwanese population to explore the association between herpes zoster and the subsequent risk of lymphoid malignancies. To the best of our knowledge, this is currently the largest matched-control study of an Asian population. Our results showed that preceding herpes zoster infection is an independent risk marker for subsequent lymphoid malignancies in Taiwanese subjects from univariate and multivariate analyses. There are several retrospective case–control studies that have explored the risks of preceding infections and subsequent lymphoid malignancies in Western populations [13-17]. Among the preceding infections, herpes zoster was shown to be associated with both Hodgkin’s disease and non-Hodgkin’s lymphoma in a hospital-based case–control study in Italy [26] and an increased risk of CLL in male U.S. veterans [13]. In a large case–control study using the U.S. SEER database, herpes zoster showed an increased risk of CLL and the risk increased with increasing severity or frequency of herpes zoster [14]. An increased risk of multiple myeloma or monoclonal gammopathy of undetermined significance was found in patients with history of herpes zoster in Italian and in white and black male U.S. veterans [15,16]. Similarly an increased risk of lymphoplasmacytic lymphoma or Waldenstrom macroglobulinemia was found from Swedish population-based registries [17].

The association of herpes zoster and subsequent risk of internal malignancies has been reviewed for decades, however most studies have not found an association between herpes zoster and an increased risk for the development of future malignancies [11,12]. In our study, the association of herpes zoster and risk of cancer was seen in lymphoid malignancies but not in non-lymphoid malignancies, which is compatible with other studies and suggests that herpes zoster is an independent risk marker for lymphoid malignancies but not a risk marker for other cancers.

Some host factors have been found to be associated with the development of lymphoma including inherited genetic factors, infections, autoimmune diseases, and environmental exposure [4]. HIV-AIDS is a known risk factor for lymphoid malignancies [1-4], and patients with HIV infection and other known viral infections that could be coded from the ICD-9-CM were excluded in our analysis to exclude the influence of viruses other than herpes zoster. Autoimmune diseases and chronic inflammatory disorders have been shown to be associated with lymphoid malignancies [27-29]. Although the proportion of autoimmune diseases in patients was higher than in the controls (4.2% vs. 2.9%), the covariates of CCI including rheumatologic diseases were adjusted in Cox proportional hazard regressions, and a relatively small difference was noted in adjusted HRs from step-wise removal of comorbidities, indicating that the influence of autoimmune diseases is low. Similarly, other systemic comorbidities may play a role in the association with the development of lymphoid malignancies. The adjustment of covariates of CCI, and a relatively small difference in adjusted HRs from step-wise removal of comorbidities, are helpful to diminish the confounding factors caused by systemic comorbidities.

The total case number of herpes zoster (n = 42,498) in our cohort before diagnosis of any malignancies is comparable with some studies focusing on herpes zoster using Taiwan NHIRD [30,31], which showed the incidence rate of herpes zoster in Taiwan is estimated around 4.89 to 4.97 cases per 1000 person-years, indicating the possibility of underdetection in our cohort is low. In addition, our dataset contained inpatient and outpatient compartments, preventing the selection and information bias common in hospital-based studies. Moreover, most of the published study designs have been retrospective case–control studies. Prospective study that shows infection preceded lymphoid malignancies and that infection is associated with subsequently elevated lymphoid malignancies risk can be especially compelling.

The distribution of lymphoid malignancies in Asian populations is different from those in Western countries [18-22]. The incidence of low grade lymphoma, CLL, and Hodgkin’s disease is lower than in Western populations, and the incidence of T cell lymphoma is higher, indicating racial or geographical differences in the etiology of lymphomagenesis. Although the cause is unclear, searching for an antecedent exposure history prior to lymphoid malignancies is helpful in determining the causes of the geographical differences. Furthermore, to better clarify the role of antecedent herpes zoster infection with subsequent lymphoid malignancies, it is important to perform analysis in a different population. Our study provides evidence supporting the association between herpes zoster infection and the subsequent development of lymphoid malignancies in Taiwanese subjects, and warrants further studies to explore the mechanism between herpes zoster and lymphomagenesis.

Many studies have attempted to explore the mechanisms between viral infections and lymphomagenesis. The potential mechanisms by which most viruses play a role in lymphomagenesis are by introducing alternations in the genome through the incorporation of viral genes, or viral infection itself may induce a state of chronic inflammation [4]. Some viral infections, like EBV, can directly infect and transform lymphocytes, disrupt normal cell function, and promote cell division [6]. The HIV infection is uinque in causing depletion of CD4+ T-cells and induces profound cell-mediated immunodeficiency which permits dysregulated proliferation of B lymphocytes. Some viral infections, like HCV, may induce chronic immune stimulation and persistent lymphocytes activation [6]. Although the mechanism between VZV and lymphomagenesis is currently unknown, the fact that the VZV establishes latency after primary infection, and herpes zoster results from a reactivation of latent VZV infection which is often associated with a decline in cell-mediated immunity [8,9] imply the possibility of the role of chronic immune stimulation. At the same time, since the decline of VZV-specific cell-mediated immunity may be a consequence of immunosuppression [10], an alternative explanation is that the herpes zoster infection is a marker of immune suppression, and such an immune suppressed condition is a potential cause of lymphoid malignancies thus herpes zoster infection is a risk marker for subsequent lymphoid malignancies. Further studies are needed to determine if VZV may trigger continuous antigenic and immune stimulation, or a disturbed immune function, and how these may be associated with the development of lymphoid malignancies.

There are limitations to our study. First, the ICD-9-CM coding system is not sufficient to classify the subtypes of lymphoid malignancies according to the 2008 WHO classification and the limited events during follow-up (n = 48), leading to difficulty in performing histological subtype analysis, although most of cases were non-Hodgkin’s lymphoma and multiple myeloma. Second, although history of previous viral infections was excluded, there were still viral infections whose compatible ICD-9-CM code could not be found, including EBV and HHV-8. However, since Taiwan is known an endemic area of EBV infection and most adults are EBV-infected [32], and HHV-8 related primary effusion lymphoma is very rare, it is likely that the influence of un-coded viral infections in patients and control group is similar. Third, the possibility of some herpes zoster infection being missed or inaccurately diagnosed exists. However, it is likely that the possibility did not differ between cases and controls, and herpes zoster has unique clinical presentations. Forth, analyzing the combined outcomes of all malignancies and lymphoid malignancies in both groups may be a limitation because there are many distinct entities with different etiologies of lymphoid malignancies. As such what we measured is an average association between herpes zoster and all lymphoid malignancies. Lastly, although several adjusted analyses had been performed to exclude the influence of medical comorbidities listed in CCI, there may be unmeasured confounding effects by other medical illness that have not been captured. The possibility that a few HIV-infected people being missed in the claims database is low because HIV-infected patients before diagnosis of any malignancies were excluded; HIV exam is an essential part when lymphoid malignancies are diagnosed; no patients were found to have HIV infection during follow-up; and HIV-infected patients have a mark in their own insurance card and they will not get the insurance coverage for HIV management if no HIV ICD-9-CM code was claimed.

## Conclusion

Preceding herpes zoster infection is an independent risk marker for subsequent lymphoid malignancies in Taiwanese subjects from a large population-based matched-control prospective follow-up survey. The role of VZV in lymphomagenesis and the possibility of chronic immune stimulation or disturbed immune function warrant further clarification. Further studies are also needed for pathogenesis exploration and preventive strategies in Asian populations.

## Competing interests

The authors declare that they have no competing interests.

## Authors’ contributions

YCL, HHH, and SFL designed the study. YCL, YHY, MYY, PML, and PYC performed the statistical analysis and interpretation of the data. YCL, HHH, WCY, TCL, CSC, and JFH drafted the manuscript. All authors contributed to the final version of the manuscript.

## Pre-publication history

The pre-publication history for this paper can be accessed here:

http://www.biomedcentral.com/1471-2407/12/503/prepub

## Supplementary Material

Additional file 1 **Table S1.** The adjusted hazard ratio of herpes zoster and the relationship with comorbidities. Click here for file
