# Supplementary material for: Herpes zoster is associated with an increased risk of subsequent lymphoid malignancies - A nationwide population-based matched-control study in Taiwan
Source: BMC Cancer. 2012 Oct 31;12:503. doi: 10.1186/1471-2407-12-503 (PMC3531246; doi:10.1186/1471-2407-12-503)
Supplement: Additional file 1 — Table S1. The adjusted hazard ratio of herpes zoster and the relationship with comorbidities. [file 1471-2407-12-503-S1.doc]

**Supplement**

**Table 1. The adjusted hazard ratio of herpes zoster and the relationship with comorbidities**

|  | Herpes zoster | |
| --- | --- | --- |
|  | Adjusted HR^a^ | 95% CI |
| All comorbidities with covariance | 1.68 | 1.19-2.36 |
| Without myocardial infarct | 1.68 | 1.19-2.36 |
| Without congestive heart failure | 1.68 | 1.19-2.36 |
| Without peripheral vascular disease | 1.68 | 1.19-2.36 |
| Without cerebrovascular disease | 1.67 | 1.19-2.36 |
| Without dementia | 1.68 | 1.19-2.36 |
| Without chronic pulmonary disease | 1.72 | 1.22-2.43 |
| Without rheumatologic disease | 1.68 | 1.19-2.37 |
| Without peptic ulcer disease | 1.69 | 1.20-2.38 |
| Without mild liver disease | 1.67 | 1.19-2.36 |
| Without diabetes (mild to moderate) | 1.68 | 1.19-2.37 |
| Without diabetes (with chronic complication) | 1.67 | 1.19-2.36 |
| Without hemiplegia or paraplegia | 1.67 | 1.18-2.36 |
| Without renal disease | 1.68 | 1.19-2.36 |
| Without moderate or severe liver disease | 1.76 | 1.19-2.36 |

^a^HR: Hazard ratio
